# Kidney Programming and Hypertension: Linking Prenatal Development to Adulthood

**DOI:** 10.3390/ijms252413610

**Published:** 2024-12-19

**Authors:** You-Lin Tain, Chien-Ning Hsu

**Affiliations:** 1 Division of Pediatric Nephrology, Kaohsiung Chang Gung Memorial Hospital, Kaohsiung 833, Taiwan; tainyl@cgmh.org.tw; 2 College of Medicine, Chang Gung University, Taoyuan 333, Taiwan; 3 Department of Pharmacy, Kaohsiung Chang Gung Memorial Hospital, Kaohsiung 833, Taiwan; 4 School of Pharmacy, Kaohsiung Medical University, Kaohsiung 807, Taiwan

**Keywords:** hypertension, oxidative stress, developmental origins of health and disease (DOHaD), nitric oxide, gut microbiota, nephron endowment, renin–angiotensin system, chronic kidney disease

## Abstract

The complex relationship between kidney disease and hypertension represents a critical area of research, yet less attention has been devoted to exploring how this connection develops early in life. Various environmental factors during pregnancy and lactation can significantly impact kidney development, potentially leading to kidney programming that results in alterations in both structure and function. This early programming can contribute to adverse long-term kidney outcomes, such as hypertension. In the context of kidney programming, the molecular pathways involved in hypertension are intricate and include epigenetic modifications, oxidative stress, impaired nitric oxide pathway, inappropriate renin–angiotensin system (RAS) activation, disrupted nutrient sensing, gut microbiota dysbiosis, and altered sodium transport. This review examines each of these mechanisms and highlights reprogramming interventions proposed in preclinical studies to prevent hypertension related to kidney programming. Given that reprogramming strategies differ considerably from conventional treatments for hypertension in kidney disease, it is essential to shift focus toward understanding the processes of kidney programming and its role in the development of programmed hypertension.

## 1. Introduction

Half a century ago, Guyton, Coleman, and their colleagues emphasized the kidneys’ role in the long-term control of blood pressure (BP) [1]. According to the Guytonian paradigm, if the kidneys fail to excrete sufficient sodium and fluid, blood volume increases, directly affecting venous return and cardiac output, which contributes to sustained high BP [2]. The kidneys release renin in response to decreased blood flow or pressure, activating the renin–angiotensin system (RAS). This activation causes vasoconstriction and further increases in blood volume, both of which elevate BP. However, while the Guytonian paradigm offers a valuable framework for understanding how kidney disease can lead to or worsen hypertension, subsequent evidence suggests that this relationship is more complex and less explored than initially thought [3,4,5].

Hypertension and kidney disease may have their roots in early life [6], as suggested by the concept of Developmental Origins of Health and Disease (DOHaD) [7]. This theory highlights how adverse environmental conditions during early development play a crucial role in shaping kidney development, ultimately leading to kidney programming [8]. These structural and functional alterations can persist and contribute to negative long-term kidney outcomes as well as hypertension. Consequently, kidney-related hypertension may arise from subtle kidney injuries that occur without any noticeable decline in kidney function, potentially even before kidney disease becomes evident. Environmental influences encompass a broad array of factors, including nutrition, maternal conditions, exposure to environmental toxins and chemicals, pregnancy complications, use of medications, infections, and external stressors [8,9].

Emerging evidence has highlighted the intricate connection between kidney programming and hypertension. Several molecular mechanisms underlying this relationship involve interactions with oxidative stress, aberrant RAS activity, inflammation, epigenetic dysregulation, dysbiotic gut microbiota, and reduced nephron number. These mechanisms associated with kidney programming reveal potential targets for preventive intervention. While hypertension and kidney disease can influence each other, this review primarily focuses on kidney programming-related hypertension. Our aim is to explore the context of hypertension linked to kidney programming and illuminate potential treatment strategies.

## 2. Kidney Programming

### 2.1. What Happens During Kidney Development?

In humans, kidney development initiates during weeks 3 to 4 of gestation and progresses throughout the remainder of pregnancy, typically completing by around 36 weeks [10]. There are three distinct kidney structures derived from the posterior intermediate mesoderm involved in this intricate process: the pronephros, mesonephros, and metanephros. The pronephros and mesonephros are primitive, temporary structures that eventually regress, whereas the metanephros develops into the definitive kidneys.

The formation and elongation of the ureteric bud (UB) initiate the development of the metanephric kidney, which then invades the surrounding metanephric mesenchyme (MM) [11]. As the MM differentiates, it gives rise to nephrons, whereas the UB undergoes branching to create the collecting ducts. The renal vesicles, formed through the conversion of mesenchyme into epithelial cells, serve as the precursors to the nephrons. The branching morphogenesis of the UB is crucial for establishing the collecting duct system, which ultimately plays a vital role in nephron formation.

The nephron is the functional and structural unit of the kidney. Nephron endowment is the number of nephrons an individual is born with, and it reflects the success of nephrogenesis. Human kidneys typically contain about 1 million nephrons, although the exact number can vary considerably between individuals [12]. Nephron development accelerates dramatically between 18 and 32 weeks of gestation, with nephrogenesis typically completing by term at around 36 weeks [13]. Following birth, the kidneys increase in size [14]. Infants typically achieve glomerular filtration rates (GFRs) comparable to adults between 6 and 24 months of age [15].

Kidney development in rodents, while similar to that in humans, occurs at a faster rate. After birth, nephrogenesis continues with the maturation of nephrons, which extends into the first 1 to 2 weeks postnatally. By embryonic day 16, both afferent and efferent nerves are present in the developing kidney [16]. These nerves infiltrate the kidney during late gestation, reach the outer cortical renal arterioles within the first 1 to 2 weeks after birth, and continue to mature postnatally [17]. Consequently, adverse prenatal and early postnatal conditions can significantly affect kidney development in rodents.

### 2.2. CAKUTs

Adverse events that occur during nephrogenesis may significantly impact kidney development, leading to kidney programming [5,7]. Notably, disrupted kidney development is associated with various clinical phenotypes, namely congenital anomalies of the kidney and urinary tract (CAKUTs) [18]. While less than 45% of CAKUT cases can be attributed to genetic factors, the majority are linked to epigenetic and environmental influences [19].

CAKUT phenotypes are often associated with a low nephron number. A deficit in nephron quantity can result in elevated glomerular capillary pressure and hyperfiltration, which subsequently leads to compensatory hypertrophy of the glomeruli. This process can initiate a harmful cycle, resulting in further nephron loss over time [3]. CAKUTs include a wide range of renal structural malformations and nephron deficits. The reduced nephron endowment often seen in CAKUTs may play a key role in the increased prevalence of chronic kidney disease (CKD) in individuals with these conditions.

CKD follows a multi-hit model [20], where a low nephron number can be seen as an initial “first hit” to the kidneys. This reduction in nephron number increases the vulnerability of the remaining glomeruli to subsequent hits, such as renal damages and environmental stressors. Accordingly, the kidneys face a higher risk of developing CKD when subjected to additional stimuli later in life.

### 2.3. Risk Factors for Kidney Programming

**Figure 1:**
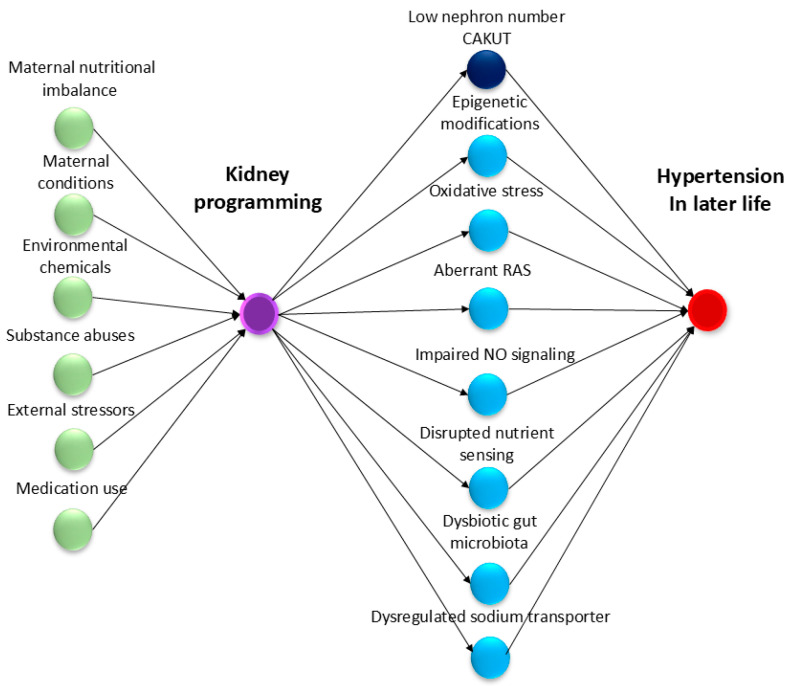
The conceptual causal model illustrates how environmental risk factors in early life influence kidney programming mechanisms, eventually leading to hypertension later in life. Black arrows indicate causal pathways, with environmental risk factors represented by green ovals and kidney programming indicated by a purple oval. Intermediate mechanisms involved in kidney programming are highlighted with a dark blue oval for morphological alterations and sky blue ovals for functional changes, while outcomes are denoted by red ovals.

A variety of environmental factors experienced during gestation and breastfeeding periods have been linked to kidney programming, possibly leading to hypertension later in life (Figure 1).

#### 2.3.1. Maternal Nutritional Imbalance

Maternal nutrition is a key environmental factor that influences organ growth and fetal development. The developing kidney is especially vulnerable to maternal nutritional imbalances. Both excessive and inadequate intake of certain nutrients have been associated with kidney programming, as highlighted in other reviews [21,22]. The Dutch birth cohort study was instrumental in establishing the notion of kidney programming, showing that insufficient nutrition during gestation has lasting effects on adult health, particularly in relation to kidney disease and hypertension [23,24]. Numerous epidemiological studies have associated maternal deficiencies in vitamin A, folate, and overall energy intake during gestation with negative impacts on the kidney function and structure of offspring [25]. Extensive evidence from animal studies highlights the influence of various nutritional factors on kidney programming, including calorie restriction [26], protein deficiency [27], high-fructose diets [28], high-fat intake [29], low-sodium diets [30], increased salt intake [30], and vitamin A depletion [31].

#### 2.3.2. Maternal Conditions

Several maternal conditions can influence kidney development and increase the risk of offspring hypertension. Maternal diabetes is one of the most extensively studied factors, disrupting nephrogenesis in both clinical and experimental models, which predisposes individuals to kidney disease and hypertension later in life [32,33,34,35,36]. For example, in a rat model of streptozotocin-induced diabetes, offspring born to diabetic mothers exhibited reduced nephron numbers, kidney injury, and hypertension [36].

Maternal obesity is another significant risk factor for CKD in offspring [34,37]. A meta-analysis has provided strong evidence that maternal obesity negatively impacts kidney development, increasing the likelihood of kidney disease in adulthood [34]. In various animal models, the offspring of obese mothers exhibited proteinuria, decline in kidney function, kidney tubular injury, and glomerulosclerosis [29,38,39].

Additionally, various models that replicate human maternal diseases and pregnancy complications have been developed to investigate kidney programming in offspring. These include conditions such as preeclampsia [40], hypertensive disorders of pregnancy [41], CKD [42], sleep disorders [43], infection [44], and uteroplacental insufficiency [45].

Hypertension affects up to 10% of pregnancies [46]. Studies using N^G^-nitro-L-arginine-methyl ester (L-NAME, a NO synthase inhibitor)-treated pregnant rats to induce maternal preeclampsia demonstrated elevated BP and kidney programming in their adult offspring [40]. In spontaneously hypertensive rats (SHRs), maternal hypertension contributes to kidney programming and subsequent offspring hypertension [41]. Furthermore, in models of maternal CKD induced by adenine, maternal uremia led to adverse outcomes in offspring, including hypertension and renal hypertrophy [42].

#### 2.3.3. Environmental Chemicals

During kidney development, many environmental chemicals can have a wide array of harmful effects on kidney health [47,48]. Some of these chemicals may disrupt nephrogenesis, resulting in a reduced nephron count and CAKUTs. Human studies have shown that maternal exposure to per- and polyfluoroalkyl substances, polycyclic aromatic hydrocarbons, phthalates, and air pollution is linked to preterm birth and low birth weight [49,50,51,52], both of which are risk factors for low nephron endowment.

Adult rat offspring born to dams exposed to di-2-ethylhexylphthalate (DEHP) exhibited hypertension and reduced kidney function, alongside the dysregulation of several nephrogenesis-related gene expressions [53]. These findings reveal that maternal DEHP exposure impairs nephrogenesis, leading to a low nephron number and subsequently increasing the risk of kidney disease and hypertension in later life [53].

#### 2.3.4. Substance Abuses

Estimates indicate that over 5 percent of pregnant women use one or more addictive substances, such as tobacco, alcohol, or illicit drugs [54]. Human studies have shown that maternal smoking during gestation is connected to fetal and infant kidney volume [55]. Similarly, evidence from animal models indicates that pregnant rats exposed to nicotine negatively impacts fetal kidney development, leading to CKD in their offspring [56,57,58]. A previous report indicated that maternal alcohol exposure adversely affects kidney function in overweight and obese children in a dose-dependent manner [59]. Furthermore, another study found that maternal alcohol exposure is related to the development of mild CKD in offspring by age 30 [60]. In a rat model examining maternal ethanol exposure, researchers noted a decrease in nephron number and kidney function in adult offspring, likely due to impaired branching morphogenesis [61]. While illicit drug use is associated with an increased risk of CKD progression [62], the influence of maternal illicit drug use on kidney outcomes in offspring remains largely unclear.

#### 2.3.5. External Stressors

Maternal stressors can adversely impact both pregnancy and the outcomes for offspring [63]. The hypothalamic–pituitary–adrenal (HPA) axis has a crucial role in the stress neuroendocrine response and can be influenced by various maternal stressors [64]. As a result, fetuses may be exposed to increased levels of glucocorticoids during periods of maternal stress or when exogenous glucocorticoids are given, potentially leading to long-lasting effects on organ development, a phenomenon referred to as glucocorticoid programming [65].

In a normal pregnancy, fetal blood glucocorticoid levels are significantly lower than those in the maternal circulation at term [65]. This difference is largely due to the protective role of the placenta, which inactivates active glucocorticoids through the enzyme 11β-hydroxysteroid dehydrogenase type 2 (11β-HSD2). Nevertheless, maternal stressors can inhibit 11β-HSD2, resulting in increased fetal exposure to glucocorticoids [66]. A recent cohort study involving 23,363 singleton-born children found that gestational glucocorticoid exposure was linked to a 1.7-fold increased risk of developing CKD over the first 10 years of life [67].

Animal studies have explored the impact of glucocorticoids on kidney development. Excessive glucocorticoids, particularly from exogenous sources, have been linked to kidney programming, resulting in a reduced nephron number. For example, in rats, repeated administration of dexamethasone during key developmental periods—specifically, gestational days 15 and 16 [68], embryonic days 16 to 22 [69], or postnatal days 1 to 3 [70]—has been associated with lower nephron numbers and hypertension in the adult offspring. RNA next-generation sequencing (NGS) analyses of the kidney transcriptome in these prenatally dexamethasone-exposed rat offspring revealed consistent alterations in 431 renal transcripts at both 1 and 16 weeks of age [71]. These findings underscore the influence of maternal stressors on kidney programming in the developing fetus.

#### 2.3.6. Medication Use

A lot of nephrotoxic drugs that risk mature kidneys can also have harmful effects when administered to pregnant women, potentially impacting the development of fetal kidneys [72]. These drugs contain, but are not limited to, aminoglycosides, cyclosporine A, angiotensin-converting enzyme (ACE) inhibitors (ACEIs) and angiotensin receptor blockers (ARBs), non-steroidal anti-inflammatory drugs (NSAIDs), antiepileptic drugs, Adriamycin, furosemide, and cyclophosphamide. Both gentamicin [73] and cyclosporine A [74] have been connected to a low nephron number, leading to kidney programming in animal models.

Aberrant activation of the RAS is implicated in kidney disease and hypertension [75], while blockading of the RAS by ACEI/ARBs is known to provide antihypertensive and renoprotective benefits [76]. Since components of the RAS are crucial for proper renal morphology during kidney development [77,78], the use of ACEIs/ARBs has been avoided in pregnant women due to the risk of drug-induced fetopathy and renal maldevelopment [79].

Another example is NSAIDs, which are commonly used by pregnant women for pain relief [80]. However, the U.S. FDA recommends avoiding NSAIDs after 20 weeks of pregnancy due to the risks of oligohydramnios and kidney damage [81]. The nephrotoxicity of NSAIDs in the fetus is related to their inhibition of prostaglandin synthesis, as these drugs can cross the placenta and impair fetal kidney development [8]. A recent cohort study involving 1,025,255 children observed that gestational exposure to NSAIDs was significantly associated with an increased risk of pediatric CKD [82]. These findings indicate that caution should be exercised when using indomethacin and ketorolac in the first trimester, mefenamic acid and diclofenac in the second trimester, and ibuprofen in the third trimester to ensure the safety of the child’s kidneys [82]. It is worth noting that the type of drug, cumulative dosage, and timing of exposure can all lead to varying effects on kidney programming.

As previously discussed, numerous environmental risk factors play a significant role in kidney programming. Given the structural changes observed, including reduced nephron numbers and CAKUTs, further research is essential to discover the underlying molecular mechanisms that drive the functional adaptations accompanying kidney programming. A thorough understanding of these mechanisms could pave the way for targeted therapeutic interventions and preventive strategies.

## 3. Molecular Mechanisms: The Link Between Kidney Programming and Hypertension

In addition to structural alterations, kidney programming involves functional adaptation through several key molecular mechanisms. Although the precise mechanisms are not fully understood, prior work has provided valuable insights into important molecular pathways involved in kidney programming. For example, factors such as epigenetic modifications, oxidative stress, impaired nitric oxide (NO) signaling, aberrant activation of the renin–angiotensin system (RAS), disrupted nutrient sensing, gut microbiota dysbiosis, and dysregulated sodium transporters have all been implicated. Figure 1 illustrates the interconnected mechanisms involved in kidney programming in response to various early-life factors, which may contribute to the development of hypertension later in life.

### 3.1. Epigenetic Modifications

Fetal programming can epigenetically influence gene expression, affecting cellular function and organ morphology, potentially increasing the risk of adult diseases like kidney disease and hypertension [83]. Key processes include DNA methylation, histone modifications, and microRNA regulation [84].

Abnormal DNA methylation of BP-related genes, such as ACE and AT1R, contributes to hypertension by upregulating components of the RAS and hypomethylating their promoters [85,86]. Additionally, histone modifications of the (pro)renin receptor and ACE, along with specific microRNAs (miR-132, miR-143/145, miR-155, miR-212), regulate BP by targeting the RAS, linking epigenetic changes to kidney disease and hypertension [87,88,89,90]. These modifications offer potential therapeutic targets for hypertension management.

Using NGS techniques, researchers analyzed epigenetically mediated gene expression in the kidneys of offspring exposed to maternal insults, identifying 809, 965, 272, and 356 differentially expressed genes (DEGs) in caloric restriction, diabetes, high-salt, and high-fructose models, respectively [91]. Notably, several signaling pathways associated with oxidative stress and peroxisome proliferator-activated receptor (PPAR) have been recognized as decisive links between kidney programming and hypertension.

### 3.2. Oxidative Stress

Oxidative stress occurs when there is an imbalance between pro-oxidants, such as reactive oxygen species (ROS), and anti-oxidant defenses. Maintaining a normal redox status is crucial for proper fetal development [92]. A variety of environmental risk factors contribute to oxidative-stress-induced kidney programming, including maternal nutritional imbalance [93], maternal disorders [94], environmental chemicals [95], substance abuse [96], and maternal stressors [97]. Various pathways of oxidative stress contribute to kidney programming, such as increased ROS-producing enzyme expression [98], increased ROS [99], decreased anti-oxidant capabilities [100], and increased oxidative damage [101].

Multiple studies have highlighted a significant connection between oxidative stress in the kidneys and hypertension, as discussed in other reviews [102,103]. Conversely, perinatal anti-oxidant administration has demonstrated protective effects against oxidative stress-related kidney programming and has also been linked to reduced hypertension in offspring across various animal models [104].

### 3.3. Aberrant RAS

In addition to oxidative stress, kidney programming is closely linked to the aberrant activation of the RAS. RAS components are highly expressed during kidney development and play a decisive role in the proper formation of renal structure and function. In animals lacking RAS genes, significant kidney malformations are observed [77]. Furthermore, blocking the RAS during nephrogenesis results in a reduced number of nephrons and an increased risk of hypertension in adulthood [105]. In human studies, the use of medications that disrupt the RAS, such as ACE inhibitors (ACEIs) and angiotensin receptor blockers (ARBs), has been linked to a higher risk of kidney malformations and ACEI/ARB-related fetopathy [72].

The classical RAS, comprising the ACE–angiotensin (Ang) II-AT1R axis, promotes vasoconstriction and contributes to hypertension [106]. Inversely, the non-classical RAS, consisting of the ACE2-Ang-(1–7)-MAS receptor axis, counteracts this effect by inducing vasodilation. Both axes of the RAS have been implicated in kidney programming [107,108]. Research shows a biphasic response: RAS expression is initially reduced at birth but normalizes with age [7]. However, this normalization in adulthood may be abnormally high, potentially leading to the inappropriate activation of the classical RAS during kidney development.

An increase in classical RAS components in the kidneys has been observed in various animal models of CKD, including the 5/6 nephrectomy/infarction model [109], the adenine-induced CKD model [110], and the streptozotocin (STZ)-induced diabetic nephropathy model [111].

### 3.4. Impaired NO Signaling

NO deficiency plays a decisive role in kidney disease and hypertension [103]. As a potent vasodilator and gasotransmitter, NO is also important for normal pregnancy and kidney development [112,113]. NO is produced through the conversion of L-arginine by nitric oxide synthase (NOS). Nevertheless, inhibition by asymmetric dimethylarginine (ADMA, a NOS inhibitor) can lead to uncoupled NOS activity, resulting in the formation of peroxynitrite and subsequent oxidative stress. Impaired NO signaling, including reduced urinary cyclic guanosine monophosphate (cGMP) levels [114], decreased L-arginine [115], reduced NOS abundance [116], elevated ADMA levels [32], and decreased NO levels [117], have all been linked to kidney programming.

In a maternal NO deficiency rat model, mother rats treated with L-NAME during gestation experienced kidney programming and offspring hypertension [114]. NO deficiency significantly altered the kidney transcriptome in neonatal kidneys, resulting in more than two thousand differentially expressed genes, most of which were associated with kidney development and epigenetic regulation [114]. Additionally, embryonic kidneys cultured in 2 or 10 µM ADMA exhibited a dose-dependent reduction in nephron number, indicating that ADMA impairs branching morphogenesis and decreases nephron formation [32]. These findings support the notion that NO deficiency contributes to kidney programming through multiple mechanisms and plays a role in its pathogenesis.

Conversely, early interventions targeting the NO signaling pathway, including supplementation with L-citrulline [68], NO donors, ADMA-reducing drugs, and enhancement of NOS abundance, demonstrate beneficial effects in mitigating kidney programming-associated offspring hypertension.

### 3.5. Disrupted Nutrient Sensing

During pregnancy, maternal nutritional status has a crucial role in coordinating maternal metabolism and fetal development through nutrient-sensing signals [118]. Accordingly, maternal nutritional imbalance can disrupt the nutrient-sensing signaling pathway concerning programmed hypertension, such as maternal high-fructose diets [119] and maternal caloric restriction [120].

Key nutrient-sensing signals that regulate maternal metabolism and fetal development include PPAR, AMP-activated protein kinase (AMPK), silent information regulator 1 (SIRT1), and PPARγ coactivator-1α (PGC-1α) [121]. Both AMPK and SIRT1 play pivotal roles in modulating metabolic processes by influencing PGC-1α [122]. AMPK activates PGC-1α through phosphorylation, while SIRT1 regulates it via deacetylation, both of which ultimately impact the expression of genes targeted by PPARs.

Several PPAR target genes participate in hypertension with developmental origins, such as *Sirt7, Nrf2, Nos2, Nos3 Ren, Sod2*, and *Sgk1* [123]. The PPAR signaling pathway is well studied in the establishment of hypertension and presents a potential therapeutic target for its treatment [124]. Previous studies demonstrated that the PPAR signaling pathway participates in hypertension of developmental origins, such as in a maternal high-fructose diet model [119] and a maternal caloric restriction model [120].

AMPK inhibition is linked to the development of hypertension [125,126], whereas AMPK activators are being investigated as potential therapies for both hypertension and kidney diseases [127,128]. Several indirect AMPK activators, including metformin, thiazolidinediones (TZDs), ginsenoside, resveratrol, berberine, α-lipoic acid, and quercetin, have shown promise in reducing hypertension related to kidney dysfunction [129]. Additionally, the direct AMPK activator 5-aminoimidazole-4-carboxamide riboside (AICAR), when administered during gestation or breastfeeding, has been found to protect male offspring from hypertension programmed by maternal diets that are high in fat [130]. This protective effect is believed to involve modulation of the AMPK-mediated nutrient sensing pathway.

### 3.6. Gut Microbiota Dysbiosis

Trillions of microbes inhabit the human gut, forming the gut microbiota, which has coevolved with humans in a mutually beneficial relationship. However, a range of environmental factors can disrupt the balance of this microbial community, leading to dysbiosis, which can have significant implications for human health and disease [131]. Maternal influences and early-life experiences have an important role in shaping the development of the neonatal gut microbiome, which gradually matures into a typical adult-like microbiota by the age of 2 to 3 years [132,133].

The gut microbiota and CKD are intricately linked in a bidirectional manner [134]. CKD can disrupt the gut microbiota, driving dysbiosis, which in turn may worsen kidney dysfunction. Conversely, dysbiosis can increase the accumulation of uremic toxins, further contributing to kidney damage and accelerating CKD progression. This dynamic interaction underscores the existence of a vicious cycle within the gut–kidney axis, where disturbances in one system exacerbate dysfunction in the other. A maternal high-fat diet has been associated with kidney programming and offspring hypertension, with alterations in the gut microbiome, including a reduction in α-diversity, an increased *Firmicutes*-to-*Bacteroidetes* (F/B) ratio, and a decrease in beneficial probiotics such as *Lactobacillus* [135,136].

Moreover, microbial metabolites play a key role in kidney programming and hypertension with developmental origins [137]. Fructose can influence microbiota-derived metabolites, such as reducing short-chain fatty acids (SCFAs) [138], increasing trimethylamine-N-oxide (TMAO) [139], and altering tryptophan metabolites [140]. Collectively, these findings suggest that fructose modulates critical microbial metabolites that contribute to developmental programming processes [141].

### 3.7. Dysregulated Sodium Transporter

Inappropriate tubular sodium reabsorption may contribute to adult hypertension induced by a suboptimal early-life environment. Upregulation of renal sodium transporters may lead to altered sodium reabsorption and hypertension related to kidney programming [142]. Alterations in the expression of specific sodium transporters were observed in a model of kidney programming induced by a maternal low-protein diet [143,144,145]. In the offspring of rats fed a low-protein diet, the mRNA expression of Na-K-ATPase subunits increased [143]. Similarly, transcriptional upregulation and higher protein levels of two sodium transporters were observed in another study of rats exposed to a maternal low-protein diet [144,145]. This study found that two key sodium transporters, the Na-K-2Cl cotransporter and the Na-Cl cotransporter, were upregulated before hypertension developed, suggesting that altered sodium handling may contribute to kidney programming-related hypertension. However, hypertension in offspring exposed to a low-protein diet is not associated with changes in renal excretory function under basal conditions [146,147]. Therefore, the role of upregulated renal sodium transporters in kidney programming-related hypertension in this model remains unclear. Additionally, increased renal expression of sodium transporters has been linked to offspring hypertension in various kidney programming models, including a maternal high-fat diet [29], prenatal glucocorticoid exposure [148], and maternal continuous light exposure [149].

Although several distinct mechanisms have been identified, they are likely interconnected, working together to drive kidney programming and contribute to the development of hypertension. A more comprehensive understanding of how these core pathways interact, as well as the discovery of additional novel mechanisms, is essential for developing effective preventive strategies to address hypertension related to kidney programming.

## 4. Targeting Kidney Programming to Prevent Hypertension

In 2020, World Kidney Day emphasized the importance of primary, secondary, and tertiary prevention of CKD [150]. Tertiary prevention focuses on managing advanced CKD and comorbidities like hypertension. Effective BP control is crucial to slow CKD progression and reduce CVD risk, with lifestyle changes such as smoking cessation, weight loss, and dietary modifications playing a key role [151,152,153]. RAS blockers remain the cornerstone of therapy for hypertension associated with kidney disease. While preclinical evidence supports the role of aberrant RAS in offspring hypertension linked to kidney programming [154], RAS blockers cannot be used for hypertension during pregnancy [155]. Many CKD patients require a combination of antihypertensive agents to achieve target BP levels [156], but only a limited number of medications are recommended for pregnant women with hypertension [155]. These findings suggest that approaches for CKD-associated hypertension cannot be directly applied to hypertension related to kidney programming.

In contrast, for kidney programming-related hypertension, primary and secondary prevention strategies are more effective. Primary prevention seeks to avert kidney disease from developing, while secondary prevention focuses on early detection and intervention to halt disease progression. The modifiable risk factors outlined in Figure 1 should be avoided during prenatal, perinatal, and early postnatal stages to promote kidney and cardiovascular health. Early detection and intervention for hypertension should begin as early as possible, even before children reach school age. However, globally, screening and diagnosing hypertension in children remains challenging due to varying definitions across different guidelines [157,158,159]. Given the discrepancies among major pediatric hypertension guidelines, there is a critical need for a global consensus to standardize clinical practices and enhance the implementation of prevention programs.

**Figure 2:**
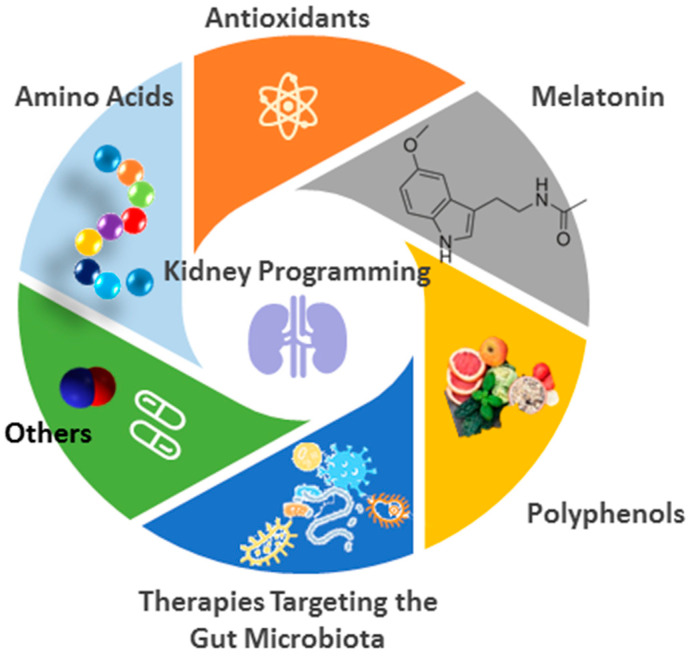
Overview of interventions to prevent kidney programming-related hypertension in animal models.

Various reprogramming strategies and interventions have been proposed in preclinical research to prevent kidney programming-related hypertension. These approaches include the supplementation of amino acids, anti-oxidants, melatonin, polyphenols, and therapies targeting the gut microbiota (Figure 2). Each of these strategies will be discussed in detail.

### 4.1. Amino Acids

Amino acids are vital for fetal programming, making perinatal supplementation a promising strategy to improve offspring health. Research has explored amino acid-based therapies, focusing on the arginine family, branched-chain amino acids (BCAAs), and methyl donors due to their influence on fetal growth [160]. While a meta-analysis supports the arginine family’s efficacy, limited studies in complicated pregnancies hinder identification of the most effective amino acids, especially for BCAAs and methyl donors [161]. Additionally, research on the long-term effects of perinatal amino acid supplementation on children remains scarce.

Amino acid supplementation as a reprogramming intervention has been studied in various kidney programming-related hypertensive animal models, including citrulline [68], cysteine [73], taurine [162], glycine [163], BCAAs [164], and tryptophan [165]. While all these amino acids demonstrated benefits in preventing offspring hypertension, they target different mechanisms of kidney programming. For instance, citrulline increases plasma arginine levels and enhances NO production by bypassing hepatic metabolism and converting to arginine in the kidneys [166]. Citrulline supplementation has revealed protective effects against offspring hypertension in rat models. In the offspring of STZ-induced diabetic dams, the perinatal use of citrulline prevented hypertension and kidney disease by modulating the ADMA–NO pathway [32]. Understanding the roles of these amino acids and translating animal findings to humans is crucial.

### 4.2. Anti-Oxidants

Given the role of oxidative stress in kidney programming and hypertension [92,103], perinatal anti-oxidant therapies are promising reprogramming strategies to prevent hypertension. Vitamins C and E, known natural anti-oxidants, have shown beneficial effects on kidney health [167]. Supplementation with vitamins C or E during pregnancy can protect offspring from hypertension induced by maternal lipopolysaccharide (LPS) exposure [168,169]. Combining vitamins C and E with folic acid and selenium also prevented hypertension in a rat model of a maternal low-caloric diet [170].

Synthetic anti-oxidants have also been used in kidney disease research, alongside natural anti-oxidants [171]. MitoQ, a coenzyme Q10 analog, prevented hypertension, nephron loss, and kidney injury in mouse offspring exposed to maternal smoking [172]. Similarly, dimethyl fumarate, a classical activator of nuclear factor (erythroid-derived 2)-like 2 (Nrf2), showed protective effects in an antenatal dexamethasone and postnatal high-fat-diet model by reducing oxidative stress markers like ADMA and 8-OHdG while enhancing NO levels [173].

### 4.3. Melatonin

Melatonin, an endogenous indolamine with anti-oxidant properties, plays a role in fetal development [174,175]. It scavenges reactive oxygen species, upregulates anti-oxidant enzymes, and increases NO bioavailability [176]. While clinically used as an anti-oxidant therapy in pregnant women and neonates [177,178], its use during pregnancy is not yet recommended despite its favorable safety profile [179].

Animal studies suggest that perinatal melatonin treatment may be a promising reprogramming intervention for kidney programming-related hypertension [180]. Models demonstrating its potential include maternal caloric restriction, maternal L-NAME exposure, a maternal high-fructose diet, and neonatal dexamethasone exposure [28,70,180]. Beyond its anti-oxidant properties, melatonin exhibits anti-inflammatory effects, regulates circadian rhythms, stimulates mitochondrial biogenesis, modulates immunity, and influences epigenetic regulation [181,182]. Despite these biofunctions, the exact mechanisms by which melatonin benefits kidney programming-related hypertension require further investigation.

### 4.4. Polyphenols

Polyphenols, plant-derived phytochemicals, offer health benefits such as anti-oxidant, antidiabetic, anti-inflammatory, anti-obesity, and prebiotic effects [183,184]. However, their limited bioavailability in vivo poses a challenge to their clinical effectiveness [185,186]. Polyphenols have been widely used in patients with kidney disease [187,188]. While polyphenol-rich diets have been shown to reduce cardiovascular risk in hemodialysis patients, their role in CKD progression remains unclear [189]. Another review of 32 studies found resveratrol to mildly protect renal function by lowering creatinine and increasing GFR, albeit with low certainty [190].

Quercetin, a prominent flavonoid, provides protective benefits against kidney disease and hypertension in offspring, as shown in rat models with maternal protein restriction and a high-fructose diet, as well as in mouse models with a maternal high-fat diet [191,192]. Additionally, several polyphenols, including flavanols, lignans, tannins, and diarylheptanoids, have shown benefits against kidney disease and hypertension in various animal models of developmental programming [193,194,195,196,197].

Among polyphenols, resveratrol is the most commonly studied stilbene. Human and animal studies suggest that resveratrol may be an effective polyphenol for preventing and treating hypertension and kidney disease [198,199]. In SHRs, perinatal resveratrol supplementation reduced the development of hypertension in adult offspring [200]. Additionally, a separate study demonstrated that perinatal resveratrol treatment protected offspring from hypertension induced by maternal CKD [201].

### 4.5. Therapies Targeting the Gut Microbiota

Currently, there is growing interest in probiotics, which are live bacteria with health benefits, and prebiotics, which are substances that promote the growth and activity of beneficial bacteria [202]. These interventions are being explored as supplements for patients with CKD [203]. However, the effectiveness of these treatments in improving kidney function in individuals with CKD remains uncertain [204].

Several gut microbiota-targeted interventions have been documented for preventing kidney programming in animal models, including probiotics, prebiotics, and postbiotics [137]. Supplementation with *Lactobacillus casei* during gestation and breastfeeding protected rat offspring hypertension programmed by a maternal diet high in fructose [205] or fat [206]. In a high-fat model, prebiotic inulin treatment prevented offspring hypertension, which was associated with alterations in the gut microbiota, particularly an increase in the abundance of the probiotic *Lactobacillus* species [206]. Acting as a prebiotic, perinatal resveratrol therapy protected against maternal CKD-induced hypertension, which was accompanied by the restoration of microbial richness and diversity, as well as an increase in *Lactobacillus* and *Bifidobacterium* species [201].

Additionally, substances released or produced through the metabolism of gut microbes, known as postbiotics, have demonstrated positive effects on the host, including in the context of kidney disease [207,208]. SCFAs like acetate, propionate, and butyrate are postbiotic metabolites that regulate BP and play a key role in kidney disease-related hypertension [209,210]. Studies show that supplementation with butyrate or propionate can prevent hypertension, potentially by increasing renal expression of SCFA receptors [211,212].

### 4.6. Others

Moreover, evidence supports the idea that early-life interventions targeting specific molecular mechanisms behind kidney programming can be beneficial in preventing programmed hypertension. First, several NO-targeting reprogramming strategies, including ADMA-lowering agents [94], NO donors [213], and enhancement of NOS expression [214], have been used in animal models to prevent programmed hypertension. Second, targeting nutrient-sensing pathways like AMPK or PPAR can regulate downstream genes and reprogram hypertension induced by various maternal insults [123,129,215,216,217].

This review provides an overview of the numerous reprogramming interventions that show promise in addressing kidney programming-related hypertension. Despite the significant progress made in animal research, clinical translation of these findings remains a long-term goal.

## 5. Conclusions

The global burden of CKD continues to grow, despite significant advances in its treatment and the management of hypertension [218,219]. While researchers are uncovering the pathophysiological mechanisms underlying kidney-related hypertension, it is crucial to shift focus toward understanding the processes of kidney programming and its links to programmed hypertension. Early identification of at-risk children and prevention strategies could make a substantial difference.

Is it time to start earlier? While substantial progress has been made in animal models of kidney programming, many challenges remain. This review synthesizes key mechanisms of kidney programming highlighted in the literature, but there is still much to explore. Specifically, further research is needed to understand how early-life environmental risk factors influence kidney programming and contribute to the development of hypertension later in life. Moreover, there is a critical need for a deeper understanding of potential reprogramming interventions, which differ significantly from those used to manage hypertension associated with kidney disease. In particular, identifying which patient populations would benefit from specific reprogramming strategies warrants serious consideration.

Future research on kidney programming should prioritize bridging the gap between animal studies and clinical applications. Investigating preventative measures and treatments targeting kidney programming-related hypertension from early life onward could lead to significant reductions in the global burden of CKD, with profound implications for public health, morbidity, and mortality.

## Data Availability

Data are contained within the article.
